# Shenqi Jiangtang Granule Ameliorates Kidney Function by Inhibiting Apoptosis in a Diabetic Rat Model

**DOI:** 10.1155/2019/3240618

**Published:** 2019-11-20

**Authors:** Qian Zhang, Xinhua Xiao, Jia Zheng, Ming Li, Miao Yu, Fan Ping, Tong Wang, Xiaojing Wang

**Affiliations:** Key Laboratory of Endocrinology, Ministry of Health, Department of Endocrinology, Peking Union Medical College Hospital, Peking Union Medical College, Chinese Academy of Medical Sciences, Beijing 100730, China

## Abstract

Diabetic nephropathy (DN) is a major microvascular complication of diabetes. In addition to moderating hyperglycemia, Shenqi Jiangtang Granule (SJG) had a beneficial effect on kidney function in a clinical trial. However, the mechanism involved remains unclear. This study was conducted to identify the underlying molecular mechanisms. A diabetic rat model was generated by using a high-fat diet and streptozotocin (STZ) injection. Then, rats were given SJG at dosages of 400 mg/kg/d or 800 mg/kg/d by gavage for 8 weeks. After 8 weeks of treatment, blood glucose, serum creatinine, blood urea nitrogen (BUN), and 24-h urinary albumin were measured. Histochemical staining and TdT-mediated dUTP nick-end labeling (TUNEL) assays were performed in kidney. Kidney genomic expression in the SJG-treated group and diabetic group was detected by using a genome expression microarray. We found that SJG treatment reduced blood glucose, serum creatinine, BUN, and 24-h urinary albumin and affected kidney histology. The gene array revealed that the expression of 99 genes increased and the expression of 91 genes decreased in the HSJG group, compared with those of in the diabetic group. Pathway and gene ontology analysis of the differentially expressed genes showed an enrichment of the apoptosis pathway. SJG treatment reduced TUNEL- and caspase-3-positive cells in diabetic kidneys. SJG upregulated Bcl-2 and regucalcin expressions and reduced casp3 and Apaf1 expressions in diabetic rats. Our results suggest that SJG exerts a renal protective effect through the inhibition of cell apoptosis in a diabetic rodent model.

## 1. Introduction

The number of patients with diabetes is dramatically increasing worldwide [1]. Forty percent of diabetic patients develop diabetic nephropathy (DN), which is a major microvascular complication [2]. In addition, DN is the most common cause of end-stage renal disease [3]. DN is also a major cause of morbidity and mortality in patients with kidney disease worldwide.

The pathological character of DN includes abnormal accumulation of extracellular matrix (ECM), thickening and hypertrophy of the glomerular basement membrane, and loss of glomerular and tubular cells, which leads to kidney fibrosis [4]. These structural changes cause increased proteinuria and albumin excretion and reduced glomerular filtration rate (GFR).

In addition to hyperglycemia, numerous pathways contribute to the development of DN, including oxidative stress [5], proinflammatory molecules [6], enhanced reactive oxygen species (ROS) [7], activation of protein kinase C [8], increased formation of advanced glycation end products (AGEs) [9], and the renin-angiotensin system (RAS).

Shenqi Jiangtang Granule (SJG) comprises Panax ginseng (*Panax ginseng C*.*A*. *Mey*), Radix astragali (*Astragalus penduliflorus Lam*.), Radix rehmannia (*Rehmannia glutinosa (Gaertn*.*) DC*.), Radix trichosanthis (*Trichosanthes kirilowii Maxim*), Fructus Schisandrae chinensis (*Schisandra chinensis (Turcz*.*) Baill*), Ophiopogonis radix (*Ophiopogon japonicus (Thunb*.*) Ker Gawl*), Fructus lycii (*Lycium chinense Mill*), Fructus rubi (*Rubus chingii Hu*), Rhizoma dioscoreae (*Dioscorea oppositifolia* L.), Alismatis rhizome (*Alisma plantago-aquatica subsp*.), and Poris cocos (*Smilax glabra Roxb*). SJG is approved by the State Food and Drug Administration of China (State medical license no. Z10950075). A clinical trial indicates that single drug treatment with SJG has an antidiabetic effect in type 2 diabetic patients, compared with that of exercise or dietary intervention [10]. A meta-analysis showed that SJG combination therapy has a better effect than traditional therapy alone, including reducing urinary albumin excretion rate (UAER), serum blood urea nitrogen (BUN), serum creatinine, and 24 h urea albumin in diabetic patients [11]. The mechanism driving this may be involved in inducing HIF-1*α* and HO-1 expression [12]. Recently, in vitro experiment revealed SJG as an alpha-glucosidase inhibitor [13]. However, the exact mechanism underlying the beneficial effect of SJG on kidney function is still unknown.

We hypothesized that SJG has multiple targets in the kidney, thus moderating the kidney fibrosis in diabetic rats. Traditional Chinese herbs usually have multiple targets. Therefore, we used a genome-wide array strategy in kidney and pathway analysis.

## 2. Methods

### 2.1. Medicine

Shenqi Jiangtang Granule includes Panax ginseng (*Panax ginseng C*.*A*. *Mey*, 0.71%), Radix astragali (*Astragalus penduliflorus Lam*, 14.71%), Radix rehmannia (*Rehmannia glutinosa (Gaertn*.*) DC*., 22.06%), Radix trichosanthis (*Trichosanthes kirilowii Maxim*, 7.35%), Fructus Schisandrae chinensis (*Schisandra chinensis (Turcz*.*) Bail*, 7.35%), Ophiopogonis radix (*Ophiopogon japonicus (Thunb*.*) Ker Gawl*, 7.35%), Fructus lycii (*Lycium chinense Mill*, 14.71%), Fructus rubi (*Rubus chingii Hu*, 3.7%), Rhizoma dioscoreae (*Dioscorea oppositifolia* L., 7.35%), Alismatis rhizome (*Alisma plantago-aquatica subsp*., 7.35%), and Poris cocos (*Smilax glabra Roxb*., 7.35%). SJG was provided by Lunan Hope Pharmaceutical Co., Ltd. (Shandong, China). The eleven component herbs, Panax ginseng (6 g), Radix astragali (124 g), Radix rehmannia (186 g), Radix trichosanthis (62 g), Fructus Schisandrae chinensis (62 g), Ophiopogonis radix (62 g), Fructus lycii (124 g), Fructus rubi (31 g), Rhizoma dioscoreae (62 g), Alismatis rhizome (62 g), and Poris cocos (62 g), were soaked in 60% ethanol for 1 h and extracted twice by refluxing for 2 h. The condensed extracts were mixed with dextrin and sugar powder to make up SJG.

### 2.2. High-Performance Liquid Chromatography (HPLC) Analysis of SJG

SJG powder (4.0 g) was refluxed 4 times by *n*-butyl alcohol. Then, the combined extracts were concentrated by vacuum-rotary evaporation. The concentration was eluted by 70% ethanol on macroporous adsorptive resin. All the collected eluents were concentrated and then dried at 40°C in a vacuum oven. Final residue of SJG was dissolved in ethanol and then filtered through a 0.45 *μ*m filter membrane. SJG was characterized by using an Agilent 1260 Infinity II HPLC (Agilent Technologies, CA, USA) with a Symmetry C18 column (150 mm × 4.6 mm, i.e., particle size 5 *μ*m, MA, USA). The column was eluted at 30°C with a detection wavelength at 203 nm and an injection volume of 10 *μ*L. The flow rate of the mobile phase of acetonitrile (A) and water (B) was set at 1.0 mL/min. Gradient separation was based on the following: 0–12 min, 0.5–5% A; 12–38 min, 5–23% A; 38–78 min, 23–40% A; 78–99 min, 40–61% A; 99–108 min, 61–80% A; and 108–120 min, 80% A.

### 2.3. Animal Treatments

Twenty-four male Sprague Dawley rats were purchased from the Institute of Laboratory Animal Sciences of the Chinese Academy of Medical Sciences and Peking Union Medical College in Beijing, China (SCXK-2014-0013) weighing 170 to 190 g each. They were placed in a 12-h light/dark cycle and 25°C controlled housing and were given food and water ad libitum. A diabetic model was generated by an intraperitoneal injection of streptozotocin (60 mg/kg, STZ, Sigma, St. Louis, MO). A week after the injection, the diabetes model was confirmed. Rats with fasting blood glucose levels greater than 11.1 mmol/L were considered diabetic. Diabetic rats were randomly divided into three groups (*n* = 6 per group): a low dose of Shenqi Jiangtang Granule (LSJG) group, a high dose of Shenqi Jiangtang Granule (HSJG) group, and a diabetic group. The typical human daily dose of Shenqi Jiangtang Granule (SJG) is 3 g per 50 kg of body weight. LSJG and HSJG were administered oral Shenqi Jiangtang Granule at 400 mg/kg/day or 800 mg/kg/day for 8 weeks. The dosage of LSJG and HSJG for rats is corresponded to 3 g/day and 6 g/day for humans.

The diabetic and control groups received an equal volume of saline. After 8 weeks of treatment, all rats were anesthetized with intraperitoneal injection of sodium pentobarbital (150 mg/kg) and then sacrificed. Kidneys were immediately removed. All animal treatment and procedures were approved by the Animal Care Committee of the Peking Union Medical Hospital Animal Ethics Committee (Project XHDW-2015-0051, 15 February 2015), and all efforts were made to minimize suffering.

### 2.4. Metabolic Parameter Analysis

After 6 h of fasting, blood was collected. Serum was isolated from the blood samples by centrifuging at 3000 g for 10 min (Heraeus Varifuge 3.0, Hamburg, Germany). Serum creatinine and blood urea nitrogen (BUN) were measured with a Beckman Coulter AU5800 analyzer (Beckman, Germany). At the end of the 8-week treatment, 24-h urine samples were collected using metabolic cages, centrifuged at 3000 g for 10 min (Heraeus Varifuge 3.0, Hamburg, Germany) and stored at −80°C. Urine albumin concentration was measured by a Beckman Counter AU5800 analyzer (Beckman, Germany).

### 2.5. Histology

Kidneys were fixed with 4% formalin and embedded in paraffin. Kidney sections (5 *μ*m) were stained with periodic acid Schiff (PAS). The glomerular damage score of each rat was calculated as the arithmetic mean of 60 glomeruli (×400 magnification) [14]. Briefly, glomerular lesions were graded as normal (or minimal) to severe (extensive damage). Severity was graded as absent/normal (grade 0), mild (grade 1), moderate (grade 2), and severe (grade 3). The tubular damage (dilation, atrophy, hyaline in tubular lumen, infiltration of mononuclear cells, and interstitial fibrosis) was assessed as previously described with a semiquantitative method from Grade 0 (normal) to Grade 3 (severe) [15]. Tubulointerstitial damage scores are given as the arithmetic mean of all fields (×400 magnification).

### 2.6. RNA Extraction and Gene Expression Array

Total RNA was extracted from the rat kidney cortex using a mirVana™ RNA Isolation Kit (Ambion, San Paulo, Brazil). Double-stranded cDNA was synthesized from total RNA. Then, cDNA was labeled with biotin. The biotinylated cDNA was purified, fragmented, and hybridized to an Affymetrix GeneChip Rat Gene 2.0 ST whole transcript-based array (Affymetrix Technologies, Santa Clara, CA). Genes were selected based on *P* < 0.05 and fold change >1.5. The data obtained have been deposited in the NCBI Gene Expression Omnibus (GEO) database (accession number GSE134071.

DAVID (Database for Annotation, Visualization, and Integrated Discovery) web-based software tool was used to perform gene ontology (GO) enrichment analysis. The Kyoto Encyclopedia of Genes and Genomes (KEGG) database was utilized to identify pathways. For interaction network analysis, the STRING (Search Tool for the Retrieval of Interacting Genes) database was used.

### 2.7. Real-Time PCR

Total RNA from the kidney cortex was used to synthesize cDNA using SuperScript II reverse transcriptase (Life Technologies, Carlsbad, CA). Quantitative PCR was carried out by using specific primers (Table 1). A SYBR Green Mix Kit (Applied Biosystems, Foster City, CA) and an ABI Prism 7500 Real-Time System (Applied Biosystems, Foster City, CA) were used for PCR. Relative expression levels were calculated with the 2^−ΔΔCt^ method.

### 2.8. TUNEL Assay

Apoptosis was detected by a terminal deoxynucleotidyl transferase-mediated dUTP nick-end labeling (TUNEL) assay, using an Apoptosis Detection Kit (Roche Applied Science, Mannheim, Germany). Briefly, paraffin-embedded kidney sections were dewaxed, washed with PBS, and incubated with Proteinase K for 20 min. After washing with PBS, sections were incubated with the TUNEL reaction mix at 37°C for 60 min.

### 2.9. Immunohistochemistry for Bcl-2 and Caspase-3 in Kidney

The kidneys were fixed in 4% paraformaldehyde and embedded in paraffin. Then, 5 *μ*m-thick sections were dewaxed. After washing in PBS, sections were incubated with 1.5% H_2_O_2_ in methanol to block endogenous peroxidase activity. Nonspecific binding was blocked with 10% normal goat serum in PBS. Sections were incubated overnight with, and rabbit polyclonal caspase-3 (1 : 50, Santa Cruz Biotechnology, Dallas, TX) at 4°C. Then, the sections were washed with PBS and incubated with a horseradish peroxidase- (HRP-) conjugated secondary antibody (1 : 2000, Santa Cruz Biotechnology, Dallas, TX) for 1 h at room temperature. After washing with PBS, the sections were stained with a 3,3′-diaminobenzidine (DAB) Color Development Kit (ZSGB-BIO, Beijing, China). The slides were analyzed using ImageJ software (National Institutes of Health, Baltimore, MD) at 400x magnification. Five slides were analyzed, and six rats were in each group.

### 2.10. Statistical Analysis

Data are shown as mean ± SD. Statistical analyses were conducted with two-way ANOVA followed by Tukey's post hoc test. Graphpad Prism 6 (GraphPad Software Inc., CA, USA) was used for data analysis. *P* < 0.05 was set for significance definition.

## 3. Results

### 3.1. HPLC Analysis of SJG

**Figure 1:**
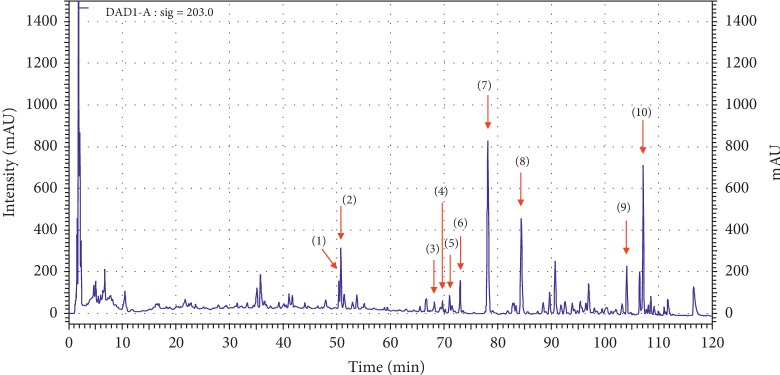
HPLC analysis of SJG. (1) Rg1 (ginsenoside Rg1), (2) Re (ginsenoside Re), (3) Rb1 (ginsenoside Rb1), (4) Rc (ginsenoside Rc), (5) astragaloside IV, (6) Rd (ginsenoside Rd), (7) schisandrin, (8) schisandrol B, (9) deoxyschisandrin, and (10) *γ*-schisandrin.

Ten main components of SJG were confirmed by HPLC analysis. The UV detector for HPLC analysis was set to 203 nm according to the standard maximum absorption rate. The HPLC analysis of SJG is presented in Figure 1. Ten main constituents of SJG are (1) ginsenoside Rg1 (15.95 mg/g), (2) ginsenoside Re (27.63 mg/g), (3) ginsenoside Rb1 (2.21 mg/g), (4) ginsenoside Rc (4.47 mg/g), (5) astragaloside IV (0.31 mg/g), (6) ginsenoside Rd (14.65 mg/g), (7) schisandrin (2.96 mg/g), (8) schisandrol B (0.75 mg/g), (9) deoxyschisandrin (0.46 mg/g), and (10) *γ*-schisandrin (1.29 mg/g).

### 3.2. SJG Showed No Influence on Body Weight in Diabetic Rats

**Figure 2:**
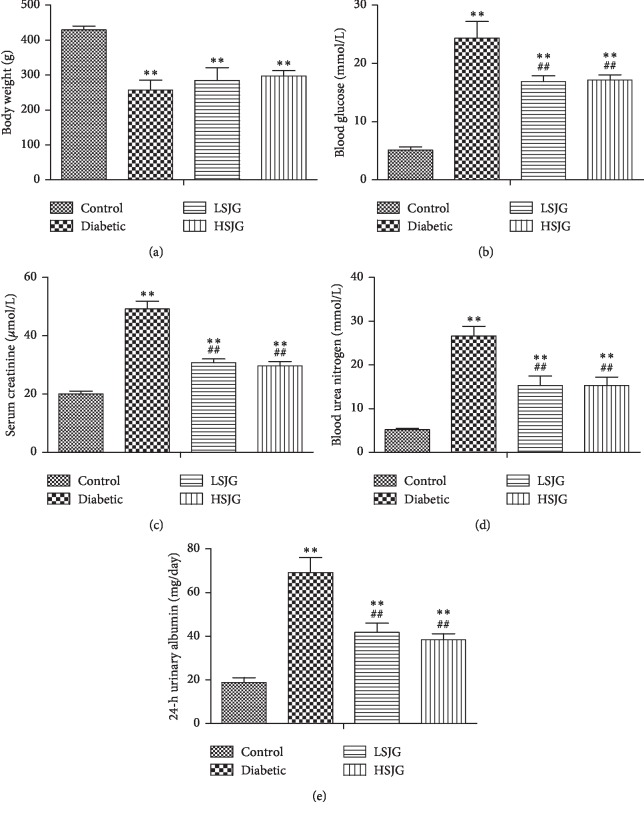
The effect of SJG on body weight (a), blood glucose (b), serum creatinine (c), blood urea nitrogen (d), and 24-h urinary albumin (e). Values are mean ± S.D. (*n* = 6), ^*∗∗*^*P* < 0.01 compared with the control group, and ^##^*P* < 0.01 compared with the diabetic group. SJG: Shenqi Jiangtang Granule.

As shown in Figure 2(a), the mean body weight of diabetic rats decreased significantly compared with that of the control rats (*P* < 0.01).

### 3.3. SJG Reduced FBG in Diabetic Rats

The diabetic rats had significantly higher FBG than the control rats (*P* < 0.01, Figure 2(b)). SJG significantly reduced the FBG in diabetic rats (*P* < 0.01, Figure 2(b)) compared with the diabetic rats.

### 3.4. SJG Moderated Kidney Dysfunction in Diabetic Rats

The serum creatinine, blood urea nitrogen, and 24-h urinary albumin increased in the diabetic group compared with the control group (*P* < 0.01, Figures 2(c)–2(e)). SJG significantly reduced serum creatinine, blood urea nitrogen, and 24-h urinary albumin (*P* < 0.01, Figures 2(c)–2(e)) compared with the diabetic rats.

### 3.5. SJG Affected Renal Histology in Diabetic Rats

**Figure 3:**
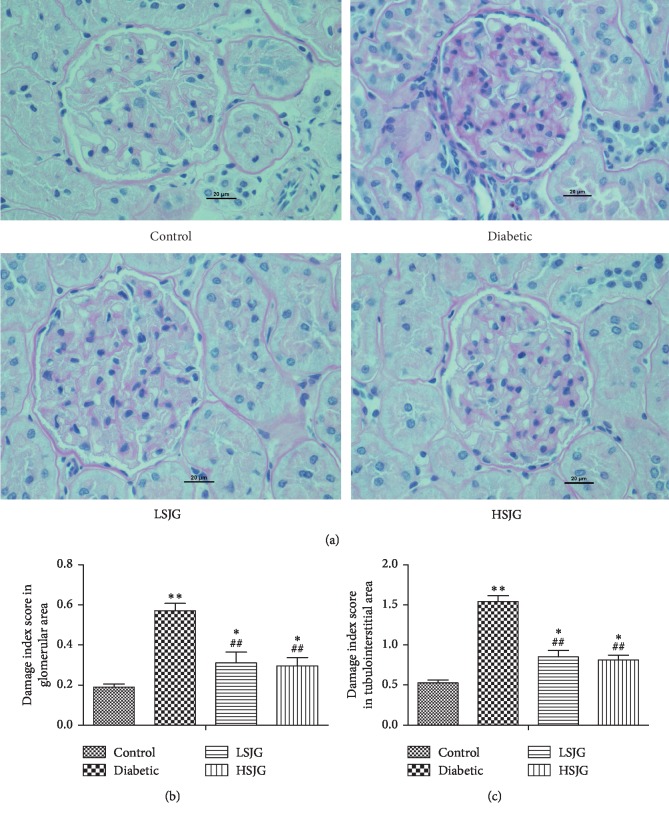
The effect of SJG on kidney histology. PAS stained kidneys (400x magnification) (a) and glomerular (b) and tubulointerstitial (c) damage index scores in kidney. Values are mean ± S.D. (*n* = 6), ^*∗*^*P* < 0.05, and ^*∗∗*^*P* < 0.01 compared with the normal group; ^##^*P* < 0.01 compared with the diabetic group. SJG: Shenqi Jiangtang Granule.

Compared with the control group, the kidneys of diabetic rats showed increased glomerular hypertrophy and tubulointerstitial changes (Figures 3(a)–3(c)). SJG treatment significantly reduced both the glomerular and tubulointerstitial changes (Figures 3(a)–3(c)) compared with the diabetic rats.

### 3.6. Gene Array Results, GO, Pathway, and Network Analysis

**Figure 4:**
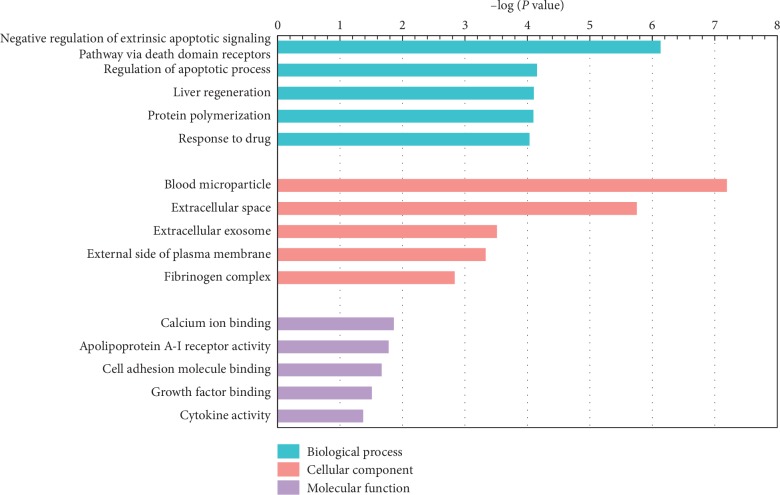
The enriched GO terms with differentially expressed genes. Top five terms in biological process (BP), cellular component (CC), and molecular function (MP).

**Figure 5:**
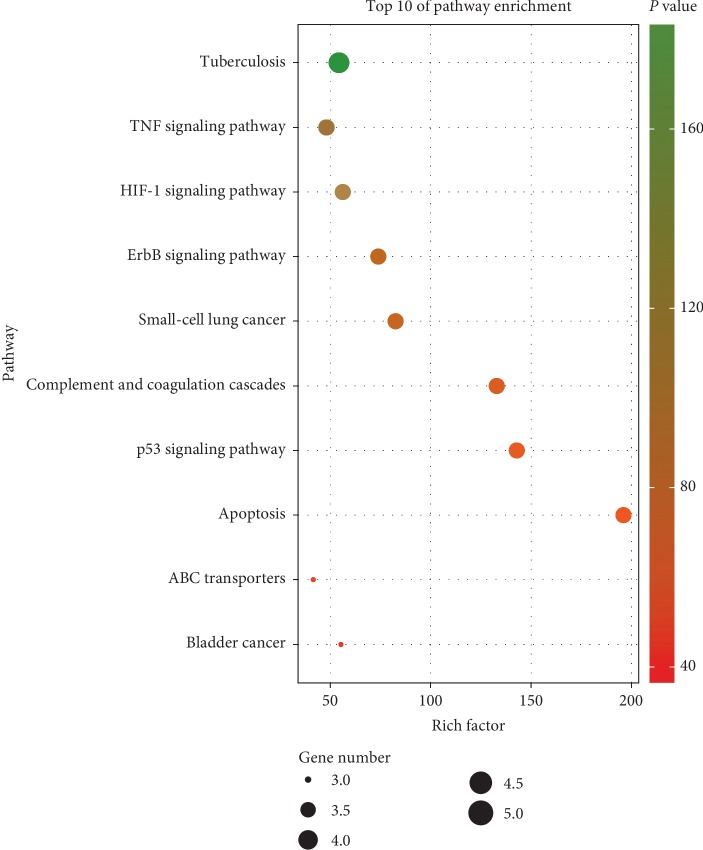
Top ten KEGG pathways enrichment point diagram in the SJG group compared with the diabetic group. The vertical axis represents the pathway name, the horizontal axis represents the rich factor, the size of the dot indicates the number of genes expressed in the pathway, and the color of the dot corresponds to the different *P* values.

Ninety-nine genes increased and 91 genes decreased in the HSJG group, compared with the diabetic group (fold change > 1.5, *P* < 0.05). To identify the relevant pathways and perform gene ontology, we analyzed the differentially expressed genes in the HSJG group vs. the control group using the DAVID. We identified significant regulation of five biological processes (BPs), cellular components (CCs), and molecular functions (MFs), respectively. Negative regulation of the extrinsic apoptotic signaling pathway via death domain receptors (involved gene number = 6), regulation of apoptotic process (involved gene number = 9), liver regeneration (involved gene number = 6), protein polymerization (involved gene number = 4), and response to drug (involved gene number = 15) were the top five terms of the BP section (Figure 4). In pathway analysis, we identified significant regulation of ten signaling pathways, including apoptosis (involved gene number = 4), p53 signaling pathway (involved gene number = 4), complement and coagulation cascades (involved gene number = 4), small-cell lung cancer (involved gene number = 4), ErbB signaling pathway (involved gene number = 4), bladder cancer (involved gene number = 3), HIF-1 signaling pathway (involved gene number = 4), ABC signaling pathway (involved gene number = 3), TNF signaling pathway (involved gene number = 4), and tuberculosis (involved gene number = 5, Figure 5).

**Figure 6:**
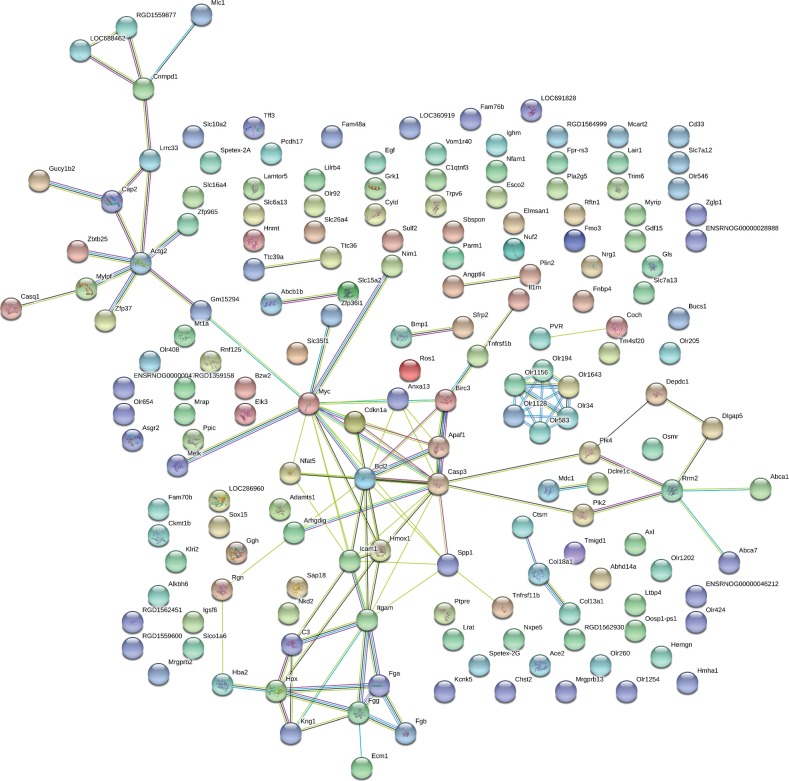
Protein-protein interaction network in the SJG group compared with the diabetic group. The nodes stand for differentially expressed genes in the SJG group compared with the diabetic group. The lines stand for the interactions between two proteins.

The 190 differentially expressed genes were mapped using the String online software. We found that 171 nodes (genes) had 114 joint edges (interactions) among these genes (Figure 6). In these nodes and edges, 11 nodes (each node with more than 5 joint edges) had 97 joint edges, representing all of the nodes. These 11 nodes are myelocytomatosis oncogene (Myc), caspase-3 (Casp3), Bcl2, integrin, alpha M (Itgam), intercellular adhesion molecule 1 (Icam1), actin, gamma 2, smooth muscle, enteric (Actg2), heme oxygenase 1 (Hmox1), apoptotic peptidase activating factor 1 (Apaf1), fibrinogen gamma chain (Fgg), hemopexin (Hpx), and secreted phosphoprotein 1 (Spp1). These genes may have important functions in kidney function affected by SJG treatment.

### 3.7. Real-Time PCR

**Figure 7:**
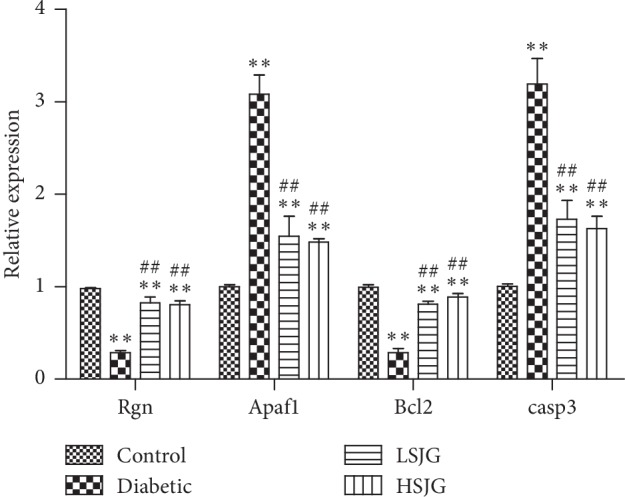
Confirmation of four representative differentially expressed genes by qPCR. Values are mean ± S.D. (*n* = 6), ^*∗∗*^*P* < 0.01 compared with the normal group, and ^##^*P* < 0.01 compared with the DN group. Rgn: regucalcin; Apaf1: apoptotic peptidase activating factor 1; and Casp3: caspase 3.

Four differentially expressed genes were analyzed using real-time PCR. We found that regucalcin (Rgn) and Bcl-2 decreased, while apoptotic peptidase activating factor 1 (Apaf1) and casp3 increased in the diabetic group, compared with the control group (*P* < 0.01, Figure 7). SJG treatment increased Rgn and Bcl-2 expression and inhibited Apaf1 and casp3 level expression (*P* < 0.01, Figure 7) compared with the diabetic rats. This result was in agreement with the corresponding data from the array.

### 3.8. SJG Reduced Cell Apoptosis in Diabetic Rats

**Figure 8:**
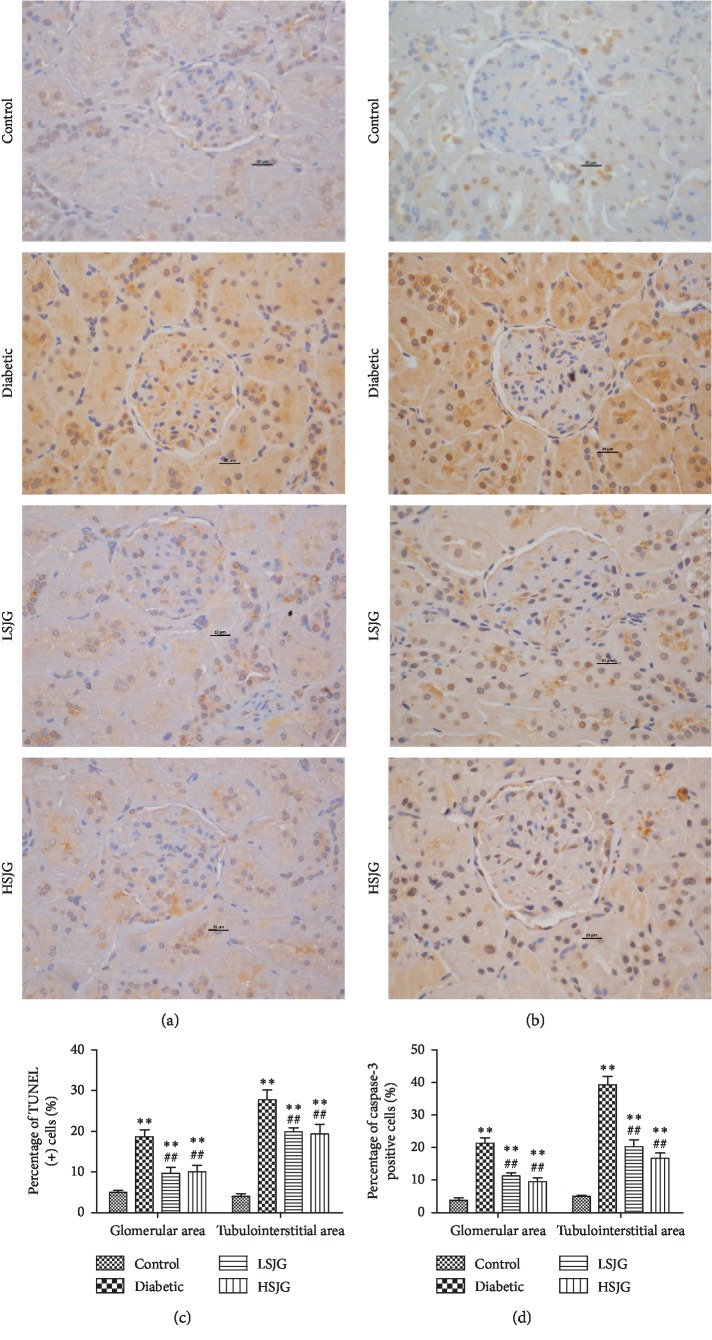
The effect of SJG on TUNEL (+) cells and caspase-3 expression. (a) TUNEL assay, (b) immunostaining for caspase-3, (c) percentage (%) of TUNEL (+) cells in kidney, and (d) percentage (%) of caspase-3 (+) cells in kidney. Values are mean ± S.D. (*n* = 6), ^*∗∗*^*P* < 0.01 compared with the normal group, and ^##^*P* < 0.01 compared with the diabetic group.

Kidneys of diabetic rats demonstrated an increased rate of glomerular and tubular cell apoptosis, as shown by TUNEL assay and caspase-3 immunostaining (Figure 8). SJG treatment reduced the rate of apoptosis in both glomerular and tubular cells in diabetic rats (Figure 8) compared with those of the diabetic rats. SJG also inhibited caspase-3 expression in diabetic rat kidney (Figure 8).

## 4. Discussion

In this study, we found that SJG treatment significantly moderated hyperglycemia in diabetic rats. A meta-analysis revealed that SJG reduced FBG and 2 h postprandial blood glucose in T2DM patients [16]. In addition, our results found that SJG could reduce serum creatinine, BUN, and 24-h urinary albumin and could moderate kidney hypertrophy and renal histology in diabetic rats. Previous studies revealed that SJG reduced urinary *α*1-microglobulin and serum cystatin in early diabetic nephropathy [17].

Shenqi Jiangtang Granule includes Panax ginseng (*Panax ginseng C*.*A*. *Mey*), Radix astragali (*Astragalus penduliflorus Lam*.), Radix rehmannia (*Rehmannia glutinosa (Gaertn*.*) DC*.), Radix trichosanthis (*Trichosanthes kirilowii Maxim*), Fructus Schisandrae chinensis (*Schisandra chinensis (Turcz*.*) Baill*), Ophiopogonis radix (*Ophiopogon japonicus (Thunb*.*) Ker Gawl*), Fructus lycii (*Lycium chinense Mill*), Fructus rubi (*Rubus chingii Hu*), Rhizoma dioscoreae (*Dioscorea oppositifolia* L.), Alismatis rhizome (*Alisma plantago-aquatica subsp*.), and Poris cocos (*Smilax glabra Roxb*). Astragalosides, which is an active ingredient of Radix Astragali (Astragalus penduliflorus Lam), has a potent antioxidative effect and inhibits high glucose-induced mesangial cell proliferation in vitro [18, 19]. Radix Astragali dramatically reduces oxidative activity in diabetic rat kidneys [19]. Two major isoflavonoids in Radix Astragali has the ability to inhibit AGE-induced endothelial cell apoptosis [20]. Radix rehmannia extract reduced BUN in STZ-induced DN rats [21].

In the gene ontology analysis of differentially expressed genes in the SJG-treated group compared with those in the diabetic group, the regulation of apoptotic process term was ranked second in the biology processes catalog. Pathway analysis of differentially expressed genes also indicated that apoptosis was one of the most enriched pathways. Moreover, TUNEL results also showed that SJG treatment reduced TUNEL-positive cells in diabetic rat kidney glomerular and tubulointerstitial areas compared with those of the diabetic rats. Apoptosis is a process of natural cell death, and it is essential for the development and normal homeostasis of all animals [22]. In the DN rodent model and in patients, hyperglycemia leads to apoptosis in various types of kidney cells, including tubular epithelial cells [23] and endothelial and interstitial cells [24]. Kidney cell apoptosis contributes to loss of kidney function [25]. Taken together, inhibiting kidney cell apoptosis following SJG treatment might be linked to kidney protective effects in diabetic rats.

In gene interaction analysis of differentially expressed genes in the SJG-treated groups compared with those in the diabetic group, Bcl-2, Casp3, and Apaf1 were centrally located within the whole gene interaction network. SJG treatment increased Bcl-2 expression and reduced Casp3 and Apaf1 expression in the kidney compared with that of the diabetic rats. Immunostaining experiments also proved that Caspase-3 expression was reduced in the SJG-treated groups, compared with the diabetic group. Mitochondrial apoptosis is regulated by a large number of proteins that directly or indirectly activate or inhibit the activity of cysteine proteases. Bcl-2 is an antiapoptotic regulator [26]. Bcl-2 inhibits the release of cytochrome c and formation of the apoptosome with Apaf1 [27], which lead to the inhibition of caspase-9 and subsequently of caspase-3 [28]. Previous research revealed that caspase-3 increases and Bcl-2 decreases in the kidneys of STZ-induced diabetic rats [29]. In immunohistochemical experiments, APAF-1 positivity was increased in diabetic tubular cells [30]. Radix Astragali is one component of SJG. In proximal renal tubular epithelial cells, Astragalosides IV, which is an active ingredient of Radix Astragali, reduced cleaved-caspase-3 expression and increased Bcl-2 expression [31]. Hence, SJG may inhibit mitochondria-dependent apoptosis to moderate kidney function in diabetic rats.

Moreover, we found that SJG activated *Rgn* expression in diabetic kidneys. Previous research has shown that regucalcin inhibits kidney proximal tubular epithelial cell apoptosis [32]. First, regucalcin causes elevation of Bcl-2 mRNA expression in rat kidney proximal tubular epithelial NRK52E cells [33]. Second, regucalcin inhibited caspase-3 expression in NRK52E cells [34]. Therefore, SJG may inhibit kidney cell apoptosis by activating regucalcin in diabetic rats.

## 5. Conclusions

**Figure 9:**
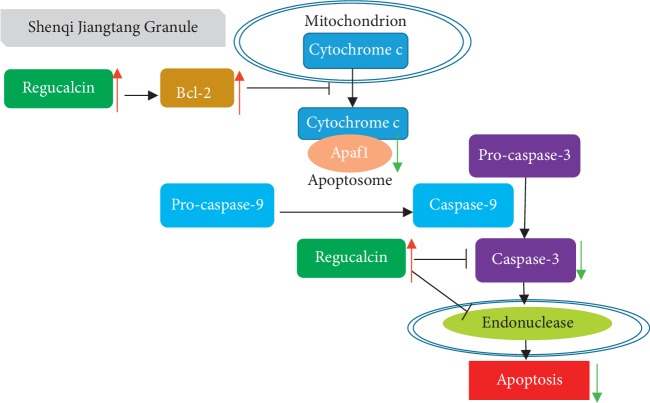
Mechanism of SJG inhibition apoptosis on the kidney in diabetic rats. SJG activates regucalcin and Bcl-2. Bcl-2 inhibits the release of cytochrome c and the formation of the apoptosome with Apaf1, which leads to the inhibition of caspase-9 and subsequent caspase-3. Apaf1: apoptotic peptidase activating factor 1.

In summary, our research found a kidney protective effect of SJG in diabetic rats. The mechanism involved may be related to the inhibition of cell apoptosis in the kidney (Figure 9). Inhibition of kidney cell apoptosis may be a potential strategy to treat DN. More experiments in vitro are needed to perform to validate this mechanism.

## Figures and Tables

**Table 1:** Oligonucleotide sequences for qPCR analysis.

Gene symbol	Gene bank ID	Forward primer	Reverse primer	Product size (bp)
Rgn	NM_031546	GGGTGGCCTGTTACAATGGA	ATCCTTCCCTCCAAAGCAGC	119
Apaf1	NM_023979	GTAGACGGCTTTCTCCGCTC	CGGATCCAGGACACAAAAGC	203
Bcl2	NM_016993	GAGGGGCTACGAGTGGGATA	CGGTAGCGACGAGAGAAGTC	243
Casp3	NM_012922	GAGCTTGGAACGCGAAGAAA	CCATTGCGAGCTGACATTCC	224

Rgn: regucalcin; Apaf1: apoptotic peptidase activating factor 1; Casp3: caspase 3.

## Data Availability

The data used to support the findings of this study are available from the corresponding author upon request.

## Abbreviations

BUN: Blood urea nitrogen
DN: Diabetic nephropathy
ECM: Extracellular matrix
GEO: Gene expression omnibus
GO: Gene ontology
GFR: Glomerular filtration rate
KEGG: Kyoto encyclopedia of genes and genomes
STRING: Search toll for the retrieval of interacting genes
SJG: Shenqi Jiangtang Granule
STZ: Streptozotocin
TUNEL: TdT-mediated dUTP nick-end labeling.
